# Appendicitis and ganglioneuroma—an unusual co-existence

**DOI:** 10.1093/jscr/rjab632

**Published:** 2022-01-22

**Authors:** Michalis Koullouros, Sarah Candler, Caroline Smith, Santosh Olakkengil

## Abstract

Ganglioneuromas are benign, fully differentiated mature tumours related to neuronal tissues and usually seen in the gastrointestinal tract, retroperitoneum and mediastinum. The few cases of appendiceal ganglioneuromas that were previously described in the literature belong to the paediatric population and were associated with genetic mutations and syndromes. We present a unique case of an Aboriginal Australian adult with acute appendicitis and concurrent ganglioneuroma diagnosed using histopathology and immunohistochemistry using Neu-N, S100 and Sox-10. The patient had no history of any of the syndromes associated with ganglioneuromatosis and had no other relevant family history.

## INTRODUCTION

Ganglioneuromas are benign, slow growing tumours arising from ganglion cells, neuronal tissues, Schwann cells or fibrous tissues [1, 2]. Ganglioneuromas were first described in 1870 by Loretz and are extremely rare with an incidence of 1 in 1 000 000 [3]. They more commonly affect females compared with males at a ratio of 3:2, and predominate the paediatric population with 60% of reported diagnoses before age 20 [4, 5]. Ganglioneuromas are part of the neuroblastic tumour group and are most commonly entirely benign with a slight potential for malignant transformation or metastasis [3, 6].

They are largely asymptomatic; however, depending upon location can present with symptoms due to compression onto adjacent structures or bleeding. For instance, those in the gastrointestinal tract can present with abdominal pain, bowel obstruction, perforation, colitis, appendicitis, anaemia, maleena or haematemesis [7].

Ganglioneuromas are associated with several genetic conditions and are rarely seen as appendiceal *de novo* solarity lesions. No biochemical markers have been correlated with diagnosis of these lesions, despite a small ratio of these producing hormones and catecholamines [8]. They have been more commonly identified in the mediastinum, retroperitoneum and colon and are most easily diagnosed after histopathological examination. Imaging modalities are commonly inconclusive despite computer tomography (CT) imaging occasionally showing low-attenuating capsulated lesions [3, 9, 10].

## CASE REPORT

**Figure 1:**
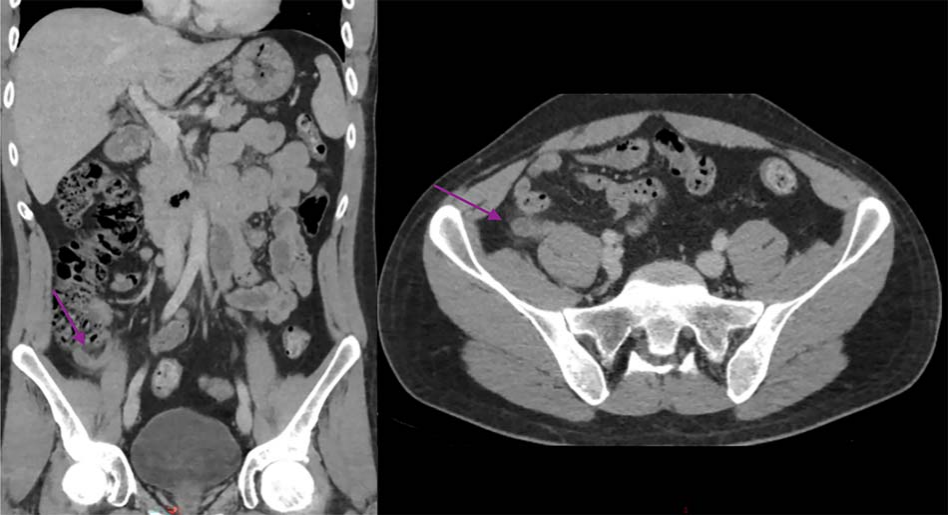
Abdominal CT scan showing a dilated and thickened appendix with periappendiceal fat stranding (arrow); coronal and axial view.

A 30-year-old Aboriginal Australian man with no past medical history presented to the emergency department with a 1-day history of lower abdominal pain. Haemodynamics were within normal range, and on examination the patient had tenderness and guarding in the right iliac fossa. Testicular exam, Rovsing and psoas signs were all negative. Blood tests revealed a mildly elevated C-reactive protein and white cells at 21.9 mg/l (0–8.0) and 11.55 × 10^9^ (4.00–11.00), respectively. Electrolytes, renal and liver function tests were all unremarkable. He proceeded to have a CT scan of the abdomen and pelvis revealing a dilated, thickened appendix with periappendiceal fat stranding. The CT also revealed uncomplicated cholelithiasis, with no other abnormalities in the bowel, adrenals, kidneys, liver, spleen or lymph nodes (Fig. 1).

**Figure 2:**
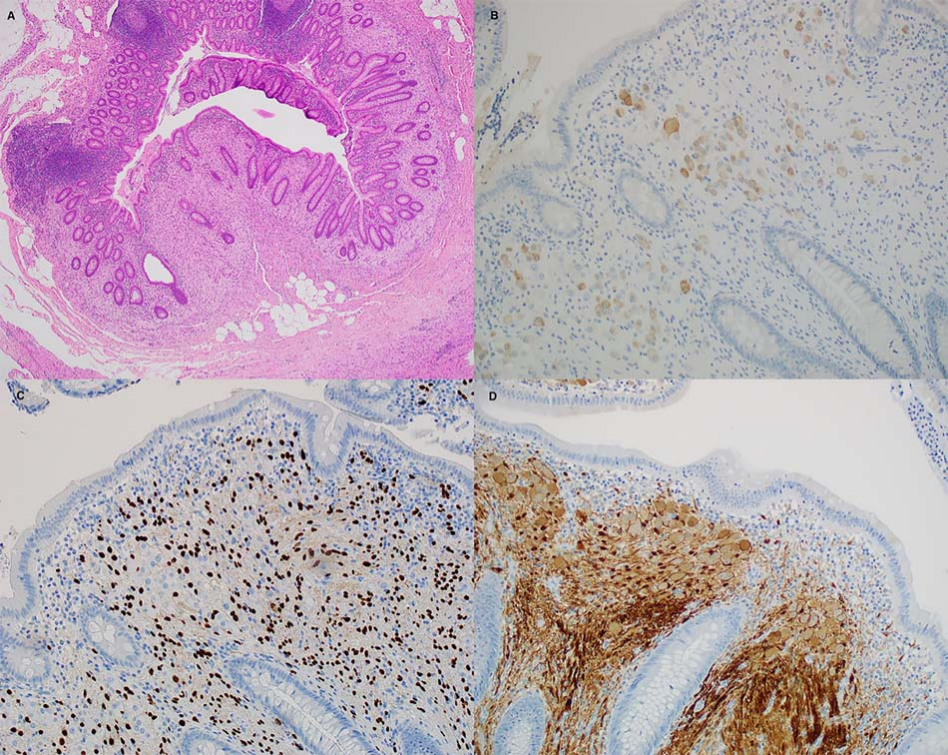
Histological sections of the ganglioneuroma. (**A**) hematoxylin and eosin, (**B**) Neu-N, (**C**) Sox10 and (D) S100.

The patient underwent a laparoscopic appendicectomy the following day. Intra-operatively the appendix appeared acutely inflamed and dilated but not perforated, and the base appeared healthy. The procedure was uncomplicated, and the patient was discharged home on Day 2 after recovering well. The histopathology revealed acute suppurative appendicitis with mucosal ulceration. Additionally, there was disordered proliferation of ganglion cells admixed with spindle cells and eosinophils at the proximal margin of the appendix, which after undergoing immunostaining for Neu-N, Sox-10 and S100 an additional diagnosis of benign ganglioneuroma was made (Fig. 2). The patient was discussed at the local general surgery multidisciplinary meeting (MDT), where a decision for no further treatment was made. The patient was counselled to seek medical opinion if he experienced any new symptoms and advised that bowel polyps are a possibility.

## DISCUSSION

Ganglioneuromas tend to be asymptomatic unless obstructing or abuting nearby structures. They usually arise from sympathetic nerve cells and, unlike the patient discussed on this report, are more often diagnosed in females under the age of 20 [4]. Rarely, they can produce catecholamines androgenic hormones or vasoactive intestinal peptides [8].

There have been cases of appendiceal ganglioneuromas described in the paediatric population, where due to significant personal and family history, the ganglioneuroma aided diagnosing genetic syndromes such as phosphatase and tensin homolog hamartoma (PTEN) syndrome [10]. Ganglioneuromas are benign; however, they are often resected when identified, as it is impossible to definitively establish their nature without histological confirmation.

On the contrary, this patient was not suspected to have a ganglioneuroma and has had no relevant personal or family history. Spontaneous solitary ganglioneuromas, such as this patient, are usually identified in the mediastinum, mesentery, retroperitoneum and colon. The lesion was not identified on cross-sectional imaging, and no other abnormalities were found either on imaging or intra-operatively. It is entirely possible that the patient will have more lesions diagnosed in the future on colonoscopy, as patients with ganglioneuromatosis are associated with familial syndromes such as multiple endocrine neoplasia type IIb, PTEN syndrome and neurofibromatosis type 1 even though there has been one report of it occurring in neurofibromatosis type 2 [9–11]. In these patients, multiple polyps are found in both upper and lower gastrointestinal tract with histology consistent with ganglioneuromas, hyperplastic polyps, hamartomatous polyps and adenomas [9].

For those reasons, we have disclosed the unusual diagnosis to the patient discussed in this report and informed them of the likelihood of similar lesions in other parts of his anatomy. It was considered pertinent to discuss the patient at the MDT meeting. Although no further treatment was considered necessary, advice was provided to the patient to seek medical attention should they experience any new gastrointestinal, hormonal or other symptoms.

## CONFLICT OF INTEREST STATEMENT

None to disclose.

## FUNDING

None to disclose.
